# Socially-Aware Adaptive Delay Tolerant Network (DTN) routing protocol

**DOI:** 10.1371/journal.pone.0262565

**Published:** 2022-01-27

**Authors:** Saif Ullah, Amir Qayyum

**Affiliations:** Capital University of Science and Technology, Islamabad, Pakistan; National Textile University, PAKISTAN

## Abstract

Network partitioning and node disconnectivity results in high latency and frequent link disruption in DTNs. Therefore, routing a message toward a destined node is a challenge in such environment. Several DTN routing schemes have been introduced in this regard. Some, recently proposed DTN routing protocols either use a single or combination of multiple social metrics to identify the suitable forwarder node(s). However, these DTN routing protocols produced results at the expense of community formation cost and over utilization of network resources. To address these issues, we propose Socially-Aware Adaptive DTN (SAAD) routing scheme which exploits a social attribute known as Degree Centrality (DC). In this scheme, each node calculates and shares its DC with other nodes at regular intervals. A forwarder node disseminates message to the most influential node possessing highest DC. The proposed routing scheme works great in situations where someone want to improve the energy efficiency and want to involve only relevant nodes. The simulation results show that SAAD has improved to select the best node and has reduced the hop-count, overhead on the expense of delay as compared to Epidemic, PRoPHET and PRoPHETv2.

## I. Introduction

Wireless networks are subject to high propagation delays, frequent disruptions and high error rate due to dynamic topology and high mobility. These limitations affect the performance of routing protocols, and it is further deteriorated in sparse and intermittent wireless environment which is known as Intermittent Connected Networks (ICNs) [1]. ICNs lack network state information (i.e., network topology and knowledge about other nodes in the entire network etc.) and thus use opportunistic approach to disseminate the messages. In an opportunistic protocol, a node possessing the data packets, forward it whenever it comes in contact with an appropriate relay node. However, if a node fails to locate an appropriate intermediate influential node, then this node has to buffer that message and carry it until it finds the desired node. This leads to another paradigm called Delay Tolerant Networks (DTNs) [2, 3]. DTN is an overlay network of intermittently connected nodes which work on store-carry and forward approach. DTN protocols can improve the efficiency of routing in partitioned opportunistic networks. Hence, DTN protocols can be exploited in the scenarios in which a message is sent to the destination in a store-carry-forward fashion [4, 5].

**Fig 1:**
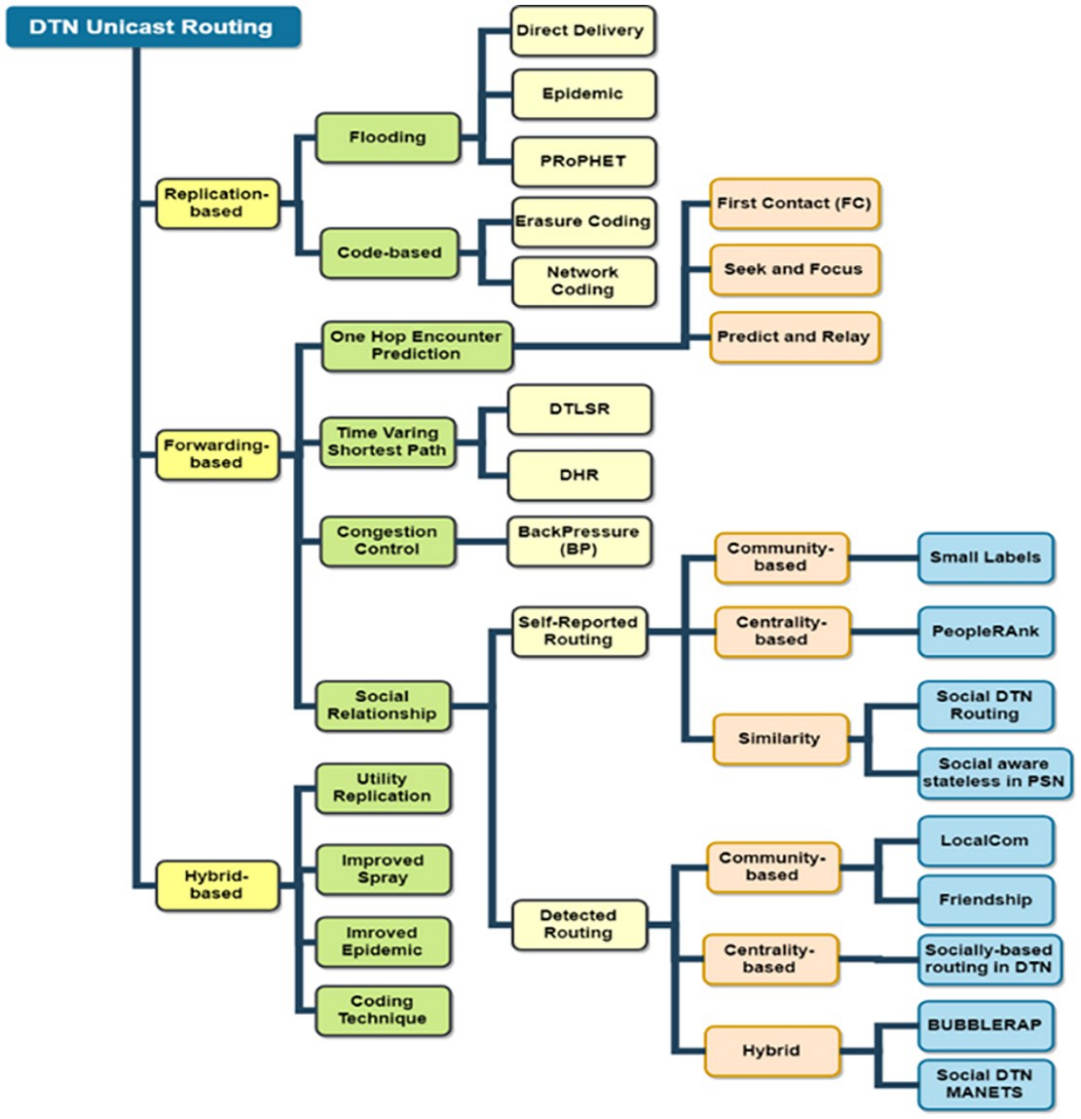
Taxonomy of DTN routing protocol.

In DTN environment, no direct link exists between a sender and receiver. Therefore, when a sender node fails to relay a message then it stores the message in its custody [6] until it finds an appropriate relay node to forward the message. Due to dynamic topology and network partitioning, DTN may be used in a variety of applications. It can be implemented to deal with post-disaster situations e.g. earthquakes, flood affected areas and emergency/rescue scenarios, where traditional networks fail to provide reliable communication between nodes, mostly because the infrastructure is itself destroyed. The existing routing protocols (i.e.; GPSR [7], AODV [8] etc.) deal with above-mentioned situations. However, recent research has proved that social relations among nodes become stable after certain period [5] which showed better delivery of packets in DTNs. Recently many routing protocols [1–5, 9–13] utilize social relations among nodes to determine when and where to forward messages. These protocols are commonly known as social-aware routing protocols. Fig 1 represents the taxonomy of the DTN routing protocols.

In this context, we would like to exploit DC to disseminate a message in an entire network. In our routing scheme, each node calculates updates and shares its DC with one-hop neighboring nodes at regular intervals. A source or intermediate node sends the packet to the most socially popular node having highest DC.

The rest of the paper is organized as follows: Section 2 describes the related work. In Section 3, we explained our proposed algorithm SAAD. In Section 4, we briefly introduce the evaluation metrics. Section 5 describes the simulation setup and results. Finally, Section 6 concludes the paper.

## II. Related work

Data dissemination is the main challenge in an intermittently connected and highly dynamic environment like in DTN. In these scenarios, a message is delivered either directly or using an intermediate potential node to its unique destination. Several routing algorithms [4–9] have been proposed in recent years which exploit social attributes.

In DTNs, three approaches are mostly followed to design routing protocols as shown in Fig 1. Firstly, a flooding technique named as “Naive Replication” in which multiple copies are sent to all encountered nodes. The second approach is named as “Utility Forwarding”, in which a packet is forwarded to other nodes based on some criteria to achieve an efficient forwarding. The third approach named as “Hybrid” is utilized by many researchers to take benefit of both the above-mentioned approaches as shown in Fig 1.

In Naive Replication approach, M. Grossglauser and D. Tse presented the Direct Delivery (DD) [14] routing technique in which a source node keeps the message in its custody till it encounters with the destination node and delivers the message directly. This routing scheme does not need any additional information about the network topology. However, to deliver a packet, direct contact between source node and destination node is necessary which results in packet loss and delay. In Two-Hop-Relay routing [14], a source node transmits a message to neighboring N nodes, who keep the message in their custody until they come in contact with the destination node and deliver the message directly which reduces delay to some extent.

In [15], Vahdat and Becker proposed Epidemic Routing to reduce delay and to increase packet delivery ratio. Pair-wise messages are exchanged to deliver a message where no end-to-end path exists between source and destination. In this technique, when a node comes across with any other node, it sends its summary vector to the neighboring nodes. The neighboring nodes then request the source node to forward only those messages which they don’t already possess in their buffer. Then the sender node transmits the messages requested by the receiving node. Each intermediate node will follow the same procedure till the message is received by the destination node. Simulation results show high message delivery at the cost of consuming large network resources (i.e., bandwidth, buffer and power).

Moreover, Spray and Wait [16] technique combines the speed of Epidemic routing in a simplest form and thriftiness of direct transmission. In this routing scheme, the message is delivered in two phases. Firstly, half of the copies of a message are sent to the network and half remained in the custody of forwarder node and wait till one of that node interacts with the destination node. This process continue still sender node left with only one copy, which it delivers to the destined node in a direct mode. Experiment-based results demonstrate that the performance of Spray and Wait scheme improves than all existing schemes in terms of number of transmissions and latency.

In Utility Forwarding, a message is forwarded to the destination node based on the network knowledge. In this strategy, the main challenge is to find the appropriate next relay node to disseminate a message based on some metrics, which may also benefit from social attributes. Now, we discuss some of the utility forwarding-based routing techniques available in the literature.

First Contact, Seek and Focus and Motion Vector are considered as One Hop Encounter Prediction-based routing. In First Contact (FC) [17], a source node sends a message to the first encountered node. FC is regarded as a one-hop encounter prediction-based algorithm in which a message is forwarded to the destination node via a number of relaying nodes. However, Seek and Focus [18] exploited random forwarding in seek phase and utility forwarding in focus phase considering the latest interaction time. Motion Vector [19], in addition to consider distance, utilizes the moving direction to calculate a new metric (geometry) value to forward a message to filter the selection process of appropriate relay node.

In Epidemic routing, messages are broadcasted to all nodes which consume more network resources. Therefore, a new routing scheme PRoPHET [20] is introduced in which a message is forwarded to an intermediate node which is selected based on some criteria (i.e.; delivery predictability and transitivity). This routing technique exploited delivery predictability (i.e.; history information of the encountered nodes) and the transitivity as a criteria to select and forward messages to the next-hop regardless of the distance. Each node calculates its delivery predictability and share with its neighboring nodes. A node having high delivery predictability and better transitivity will be considered the most appropriate forwarder node. In this routing scheme, the message can be sent to more than one node having the same delivery predictability.

In Peoplerank [21], social relationship is used to assign rank to the nodes. Whenever two nodes encounter in a social network, they share two types of information with each other, i.e. own Peoplerank value and their neighbor’s People rank value. A node which is socially connected with other high ranked nodes in a network is considered as high Peoplerank node. Messages are propagated from a low Peoplerank node to high Peoplerank node till the message reaches to the destination. It has been evaluated that Peoplerank algorithm improves delivery rate which is closed to the epidemic and reduced up to 50% retransmissions.

In a socially-based routing protocol for delay tolerant networks [22], a social-based routing scheme is used to solve limited storage space and power issues. This routing scheme exploits social criteria to select a relay node to decrease the no of copies and retransmissions. When the nodes interact with each other, they update degree of connectivity and exchange data packets which they don’t possess in their buffer. The results reveal that the routing scheme reduces the number of transmissions which saves network resources while maintaining same or high delivery ratio.

In Friendship [23], authors are convinced that friends are far better choice to select a forwarder node. Therefore, they suggested a new attribute known as Social Pressure Metric (SPM), that is exploited to measure direct friendship based on node’s history. They also proposed Conditional Social Pressure Metric (CSPM) value of its friends to find indirect friendship relationship. Once a node constructed its friendship community at a given interval, the message is transmitted using a forwarder node which must be a part of the friendship community of the destination node. The results demonstrated that the proposed scheme provides better delivery but at the cost of community formation overhead.

In [24], the authors exploited three social features (i.e. Similarity, Activeness and Centrality) to find the high priority neighboring node as a next-hop. Using Fuzzy logic, each node in the network computes the priority of its neighbor node. The numeric value of these three social features (i.e. Similarity, Activeness and Similarity) is converted into fuzzy value to calculate the priority of its neighbor node. FCSA enhanced delivery ratio and reduce end-to-end delay as compare to the existing routing schemes but they use social attributes partially only on intersections.

In Social Acquaintance-based Routing Protocol (SARP) [25], the acquaintance of local and global community nodes is considered to overcome the shortcomings of conventional routing protocols. SARP calculates the priority value of a node by combining all three social attributes (i.e. social acquaintance, social activeness, and DC). However, members having global knowledge of community are far better in intercommunity communication. For message dissemination, an intermediate node is selected based on local and global knowledge of a community member. Secondly, along with community acquaintance, a member’s activeness and similarity will also be considered. SARP improves packet delivery ratio and end-to-end delay as compare to AODV and GPSR.

In EpSoc [26], authors introduced a hybrid approach which uses the flooding strategy and utilizes a significant social feature called DC. Two different approaches are used in EpSoc to improve the performance of a routing in the opportunistic mobile social network (OMSN). Firstly, TTL value of a message is adjusted based on the DC of nodes. Secondly, the replication is controlled using blocking mechanism. Simulation results indicates that EpSoc enhances the packet delivery ratio, reduces the average latency, overhead and hop counts as compared to Bubble Rap and Epidemic.

In SimBet [27], two social attributes (i.e.; betweenness centrality and similarity) are exploited to find a forwarder node. This routing technique routed a message to a more central popular node when destination node is unknown to the sending node or intermediate nodes. Simulation results using real trace data showed almost the same delivery ratio to Epidemic but with reduced overhead. Additionally, SimBet routing scheme performs better than PRoPHET, particularly when the sending and receiving nodes have low connectivity.

In LocalCom [28], a community is constructed using limited local information and improves the forwarding efficiency based on the community structure. The encounter history of nodes is exploited to detect Similarity between each pair of nodes. LocalCom routing technique improves the delivery ratio, especially with a moderate delay requirement as compare to PRoPHET and Bubble Rap.

In Bubble Rap algorithm [29], two social metrics (i.e.; centrality and community) are used to select the most influential relay node which enhances delivery performance. Simulation results show that this routing scheme produced similar delivery ratio to flooding, control flooding, PROPHET, and SimBet but with reduced resource utilization [30].

PRoPHETv2 [31] is the updated version of the old PRoPHET in which minor modifications are made in the metric (i.e.; delivery predictability (DP)) calculation. The main objective of this routing scheme is to improve the performance of existing PRoPHET. The problem in the previous transitive equation was that as long as β > 0, the DP for every known node k will increase regardless of whether any node in the network has recently met node k or not. Although these transitive increments are usually small, they will inappropriately raise the DP values in the case of a higher encounter frequency (for nodes other than k). When this fake information spreads in the area where a high encounter frequency is observed, the problems can be expected. As a solution to this problem, we propose a new transitive update equation that does not have the same additive property as the original equation. Instead, we compare the old DP value P(A, i)old and a product of P(B, i)×P(A, B)×β values (where we suggest the default β value to be set to 0.9) and then choose the maximum value as a new DP value P(A, i). P(A, i) = max(P(A, i)old, P(B, i)P(A, B) × β) By taking the maximum. We allow for the evolution of the DP value without subjecting it to exaggerated growth when no new information is available.

The strengths and weaknesses of the DTN routing protocols which are exploiting social metrics are summarized as shown in Table 1.

**Table 1:** Summary of social metrics-based DTN routing protocols.

Articles	Characteristics of Social Ties	Strengths	Weaknesses
FCSA [24]	Degree Centrality, Similarity, Activeness	Enhance packet delivery ratio and reduce average delay	use social attributes only on intersections
Local Com [28]	Similarity	Control redundancy and tradeoff between latency and delivery ratio	Community cost, number of forwards larger than Bubble Rap
Friendship [23]	Friendship	Better delivery rate	Community formation cost
SUDS [5]	Friendship, Interest Centrality	Better data dissemination	Zone formation overhead, Infrastructure set up cost
SimBet [27]	Egocentric-based Betweenness Centrality, Similarity	Delivery performance is close to Epidemic with low overhead	-
Bubble Rap [29]	Centrality, Community	Similar delivery ratio with minimum resource utilization	Low scale
PeopleRank [21]	Social interaction frequency (centrality)	Reduce retransmissions upto 50% compared to Epidemic Routing	Unlike PageRank, it can be implemented in a centralized way but in distributed fashion.
SARP [25]	Community knowledge, Social Activeness, Degree Centrality	Improve delivery ratio	Community cost
EpSoc [26]	Degree Centrality	Improve delivery ratio, overhead and hop counts	-

After extensive literature review of the DTN routing protocols, we analyzed that although naive replication-based routing protocols provide high delivery ratio in some scenarios but utilize high network resources. The forwarding-based routing protocols use some criteria to select an appropriate relay node. These protocols either use attributes like distance, location or social attributes to find the most popular node(s) in its surrounding. Social attribute-based routing protocols have shown better results comparatively because social relations among nodes are more stable after a certain period of time.

In recent years, a number of social-aware routing protocols have been proposed to enhance the performance of different social networks in terms of maximizing the throughput and to minimize end-to-end delay. However, concerning DTN, very few routing schemes are available in literature. Most of them incurs high community cost, high bandwidth and overhead ratio. Considering the importance of social aspects connected with DTN in future, we are interested to contribute in DTN routing domain to further improve the performance of DTN.

## III. Proposed routing protocol

The objective of our proposed Socially-Aware Adaptive DTN (SAAD) routing protocol is to minimize the resource utilization and increase packet delivery in DTN by using the appropriate forwarder node(s). The proposed routing scheme exploits a significant social attribute, DC, to find the most popular and central node.

In SAAD, the process of routing a message is divided into two phases e.g. calculation of DC and message forwarding. In first phase, whenever a node joins the network, its DC (the total number of connected nodes) is calculated. This DC value of a node can be increased or decreased due to the joining/leaving nodes in the network due to high mobility in DTN. This DC will be used in the next phase for the selection of the forwarder node.

In second phase, source node will compare its DC value with all other nodes which lies in the transmission range. Only those nodes will be shortlisted which has higher DC as compared to source node’s DC and also greater than threshold value (which is discussed in the following paragraph). Shortlisted nodes are sorted in the descending order with respect to their DC. After that, source node will select first node from the sorted list which possess highest DC and forwards message (m) to the selected node. This process will continue for a given time period *t*. At the end of simulation period, a statistical report is generated which contains message delivery, overhead ratio, hop count etc.

**Fig 2:**
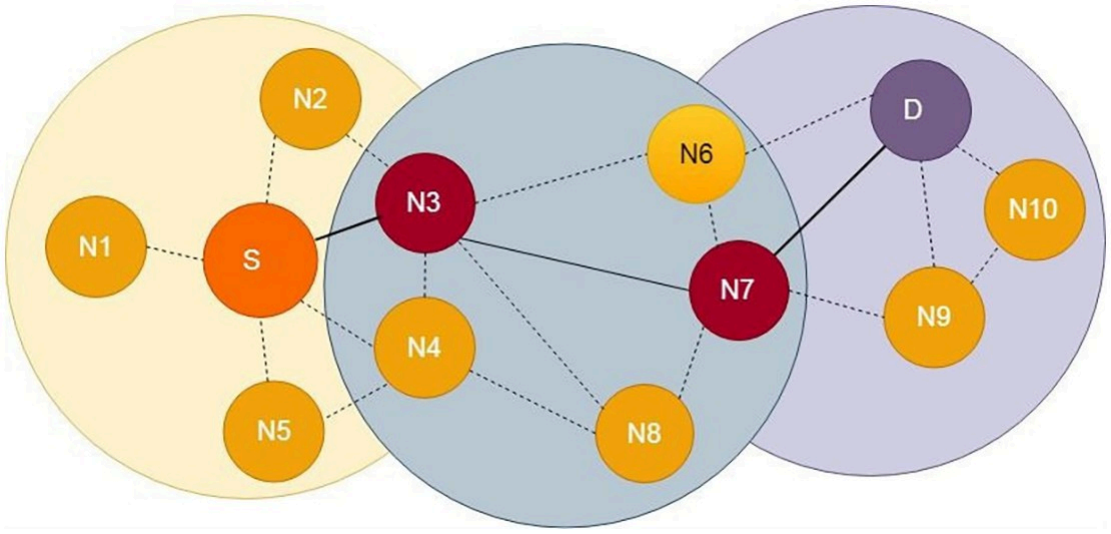
Appropriate forwarder node selection.

In literature, threshold value is being used to achieve different goals. For example, Kang et al. [32] used delivery predictability threshold value to control the speed of message dissemination. Similarly, we used threshold value in this work to achieve different goals. First, with the help of threshold value, broadcasting is controlled by selecting only those relay nodes which have higher DC value as compared to threshold value. As a result, network resources are saved e.g. bandwidth, computing power etc. With fewer nodes, more messages can be delivered successfully as messages drop ratio gets low. The process of selecting the most appropriate forwarder node is shown in Fig 2.

**Fig 3:**
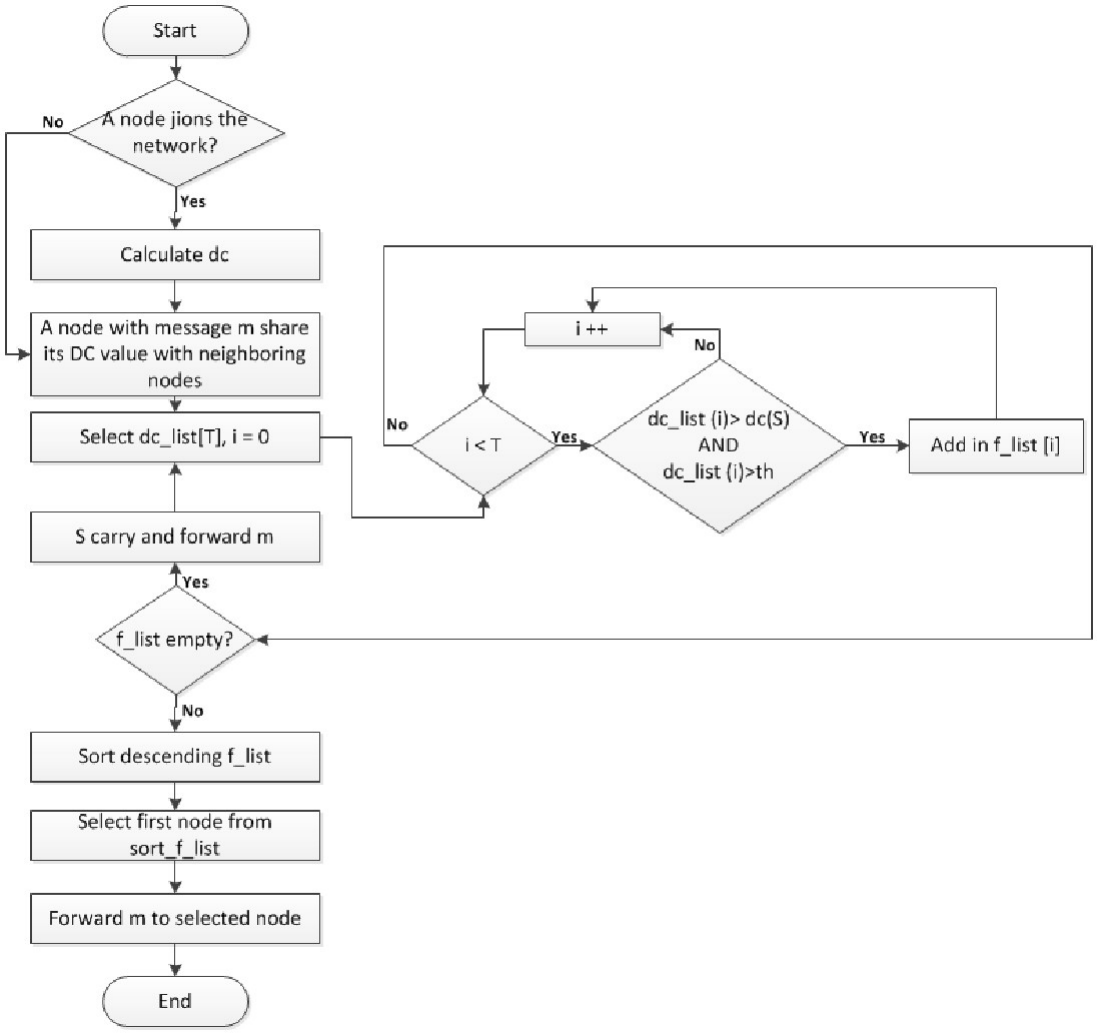
Operation flow of SAAD routing scheme.

Fig 2 demonstrates that in DTN environment, a source node wants to send a message to a destination node. Each node’s DC is calculated whenever a new node joins the network and shortlists (f-list) the nodes possessing DC value greater than the source node and threshold value in descending order. Then from the sorted shortlist (sort-f-list) selects the relay node having highest DC and forwards the messages to node N_3_ which possesses highest DC. The same process will be followed by node N_3_ and forwards messages towards the node N_7_. Finally, destination node is in radio range of N_7_, so N_7_ directly forwards message to destination node. The flow of selecting a forwarder node is also shown below in Fig 3.

**Fig 4:**
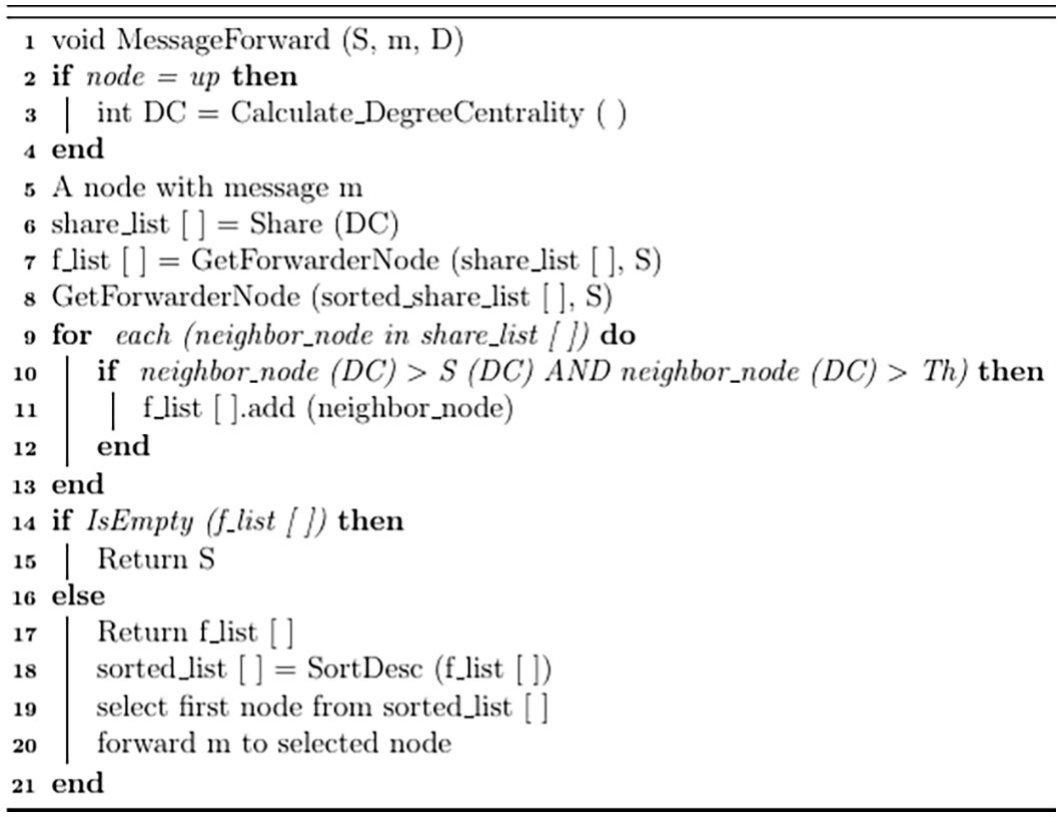
Pseudocode of our proposed routing scheme (SAAD).

Whenever a node joins the network, the new node and only those nodes which come in its range will recalculate their DC values according to the node connectivity. A source node which has a message to be sent, will share its DC value with its neighboring nodes and will obtain DC values of its neighboring nodes. Source node will check the DC value of all its neighboring nodes. For each neighbor node, two conditions will be checked. First, DC of selected node should be greater than source node. Second, DC of selected node should also be greater than threshold value. Only those nodes will be shortlisted (f_list) which meet both conditions. If f_list is not empty, f_list is sorted in descending order according to their DC values (sorted-f-list) and source node forwards the message to the first node from sorted-f-list. However, if f_list is empty, then source node will carry message itself. The corresponding pseudocode of our proposed routing scheme is shown in Fig 4

### Degree Centrality (DC)

DC [26] is calculated based on the direct links of a node in its transmission range. DC of the nodes is calculated using the following Eq 1.

$${DC}_{i}\;\left(t\right)=\sum_{K=1}^{N}\left(Cij\right)$$ (1)

Where *C*_*ij*_ = 1 means *node*_*i*_ and *node*_*j*_ have direct communication link with each other. DC is updated periodically.

## IV. Performance evaluation

### A. Metrics

The performance of the proposed SAAD routing scheme is evaluated by comparing its results with other routing schemes based on varying random seed and buffer size of nodes. Brief description of the performance metrics is given below:

#### Packet Delivery Ratio (PDR)

Packet delivery ratio is calculated by computing the number of successfully received packets to the total number of packets sent by source. The higher delivery probability means more messages are delivered to the destination successfully. This is the most important metric to evaluate the performance of a routing schemes.

#### Overhead ratio

Overhead is calculated by measuring the ratio between total number of message relayed and total number of messages delivered. The lower overhead means less utilization of network resources e.g. storage space, bandwidth etc.

#### Average end-to-end delay

The average time used to successfully deliver all messages from sender to receiver. Low average end-to-end delay shows better routing performance.

#### Average hop count

Hop count is the total number of intermediate devices such as routers through which a message is forwarded from a source to destination. Low average hop count indicates fast message delivery which overcome the network overhead.

### B. Comparison

We selected the following routing algorithms to compare the results of our proposed routing scheme.

The following routing schemes are available in ONE simulator.

#### Epidemic routing

This routing scheme used a flooding technique in which a message is sent to all neighboring nodes. This process continues till the message reached to the destination. This technique uses more network resources because of flooding. However, this routing scheme is considered to be the most important flooding technique in DTN.

#### PRoPHET

This is the first routing technique in which a message is sent to the destination using intermediate nodes which are selected based on some criteria. This technique used delivery predictability and the transitivity to select and forward a message to the next-hop. In literature, many routing algorithms compared their results with PRoPHET as it is considered to be the first routing technique which exploits metric to select a forwarder node.

#### PRoPHETv2

This is the updated version of the old PRoPHET in which minor modifications are made in the metric calculation. The key objective of PRoPHETv2 scheme is to increase the performance of existing PRoPHET.

## V. Simulation setup

To evaluate the performance of SAAD, number of simulations have been performed while taking into account disaster scenario. We used ONE (Opportunistic Network Environment) [33] simulator for our proposed routing scheme. For simulation scenario, we assumed an area of 3000 x 1500 m^2^ in disaster site. We consider the Random Waypoint model to track the pedestrians as this mobility model is commonly used in evaluations of DTN routing schemes. In this model, each node selects a random destination and starts its movement. When a node reaches at the destination, the node pause for a while and then selects a new destination. This process continues till the simulation time ends. The important parameter values which are used in default setting file for simulation are shown in Table 2.

**Table 2:** Simulation parameters.

Parameters	Value
Network Simulator	ONE
Simulation time	12 hrs.
No of nodes	50
Simulation area	3000 x 1500 m^2^
Traffic source/destination	Random Selection
Mobility model	Random Waypoint
Buffer size	5MB, 10MB, 15MB, 20MB, 25MB
Speed	0 m/sec, 20 m/sec
TTL	300 mins
Transmit range	100 m
Transmit speed	2 Mbps

To assess the performance of the proposed routing scheme, we uses various values for the parameters (e.g. speed, time to live (TTL), buffer size etc.) to get random results of the simulation. We use TTL value sothat a packet should not move infinite time in the network. In literature, TTL value is used in minutes as well as number of hops. However, ONE used TTL value in minutes. After each update interval, TTL value is reduced. When TTL value reaches to ‘0’. Nodes which possess this packet will discard it immediately. We use various buffer sizes (i.e; 5MB, 10MB, 15MB, 20MB and 25MB) and random seed values (i.e; 30, 50, 100, 150 and 200). As we increase buffer size, the more messages can reside in buffer long enough to be delivered to the destination. The ratio of drop packets also decreases as packets drop at smaller queue size. In our scenario, a warm up period of 500 seconds is set so that the process of calculating DC initializes before any message is generated. We assume that all nodes in the network are fully cooperative for fair implementation and simulation of our scheme.

## VI. Results and discussion

With the help of simulation setup mentioned-above, simulations have been performed for SAAD and three other routing schemes (i.e., Epidemic, PRoPHET and PRoPHETv2). This section discusses the comparison of SAAD results with above-mentioned routing techniques. Results are compared in terms of evaluation metrics mentioned in section-IV.

**Fig 5:**
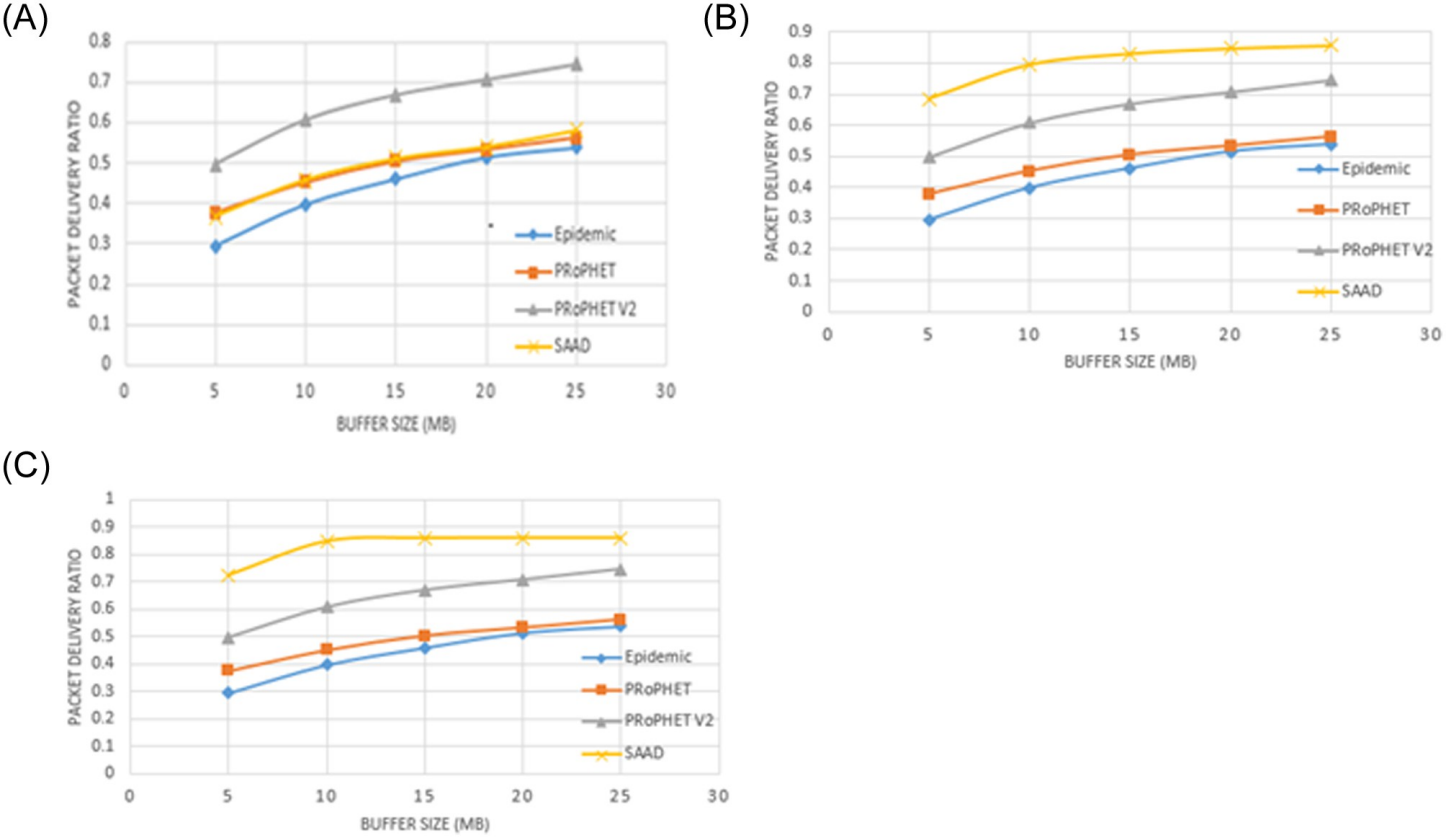
A. Packet Delivery Ratio with different buffer capacity and DC (threshold = 0). B. Packet Delivery Ratio with different buffer capacity and DC (threshold = 2). C. Packet Delivery Ratio with different buffer capacity and DC (threshold = 4).

Graphs shown in Fig 5A to 5C show the message delivery ratio. Various buffer sizes used for the simulation. For each buffer size, simulation has been run five times (12 hours each) and then average of the five simulations is taken as a final value. It depicts that as the queue size increases, more massages can reside in a buffer and hence more messages are delivered to their destination. Another important point is that as we increase the threshold value, message delivery is improved because fewer nodes are shortlisted which delivered more massages. Ultimately, less bandwidth is utilized.

We start simulation with threshold value = 0 so that a node even if it has a single neighbor node, can participate in the message forwarding process. When we use threshold value ‘0’, our proposed scheme performed bit lower than PRoPHETv2 as shown in Fig 5A because large number of nodes are initially listed which reduces the performance of SAAD in terms of message delivery. However, SAAD performs better than Epidemic and PRoPHET.

In Fig 5B, all nodes that have a DC value greater than 2 or more are considered relay nodes. In this scenario, less number of nodes are initially shortlisted as compare to the previous scenario which increases the performance of SAAD to some extent in terms of message delivery. Therefore, as we increase the threshold value to 2 and 4 respectively, message delivery increases as shown in Fig 5B and 5C.

When we use the speed of nodes (0 m/sec, 10 m/sec), we observed that packet delivery ratio was low while we increase the speed of nodes (0 m/sec, 20 m/sec), packet delivery ratio increase to some extent. However, changing the values of buffer showed a larger impact on the delivery ratio, overhead ratio, hop-count and average latency.

We analyzed the impact of buffer capacity on the simulation results, resultantly, as we increase the buffer capacity (i.e; 5MB, 10MB, 15MB, 20MB, 25MB, etc.); the delivery ratio and average packet delay are also increased. The proposed routing scheme has shown a significant improvement in packet delivery ratio as well as in overhead at the cost of longer delays.

**Fig 6:**
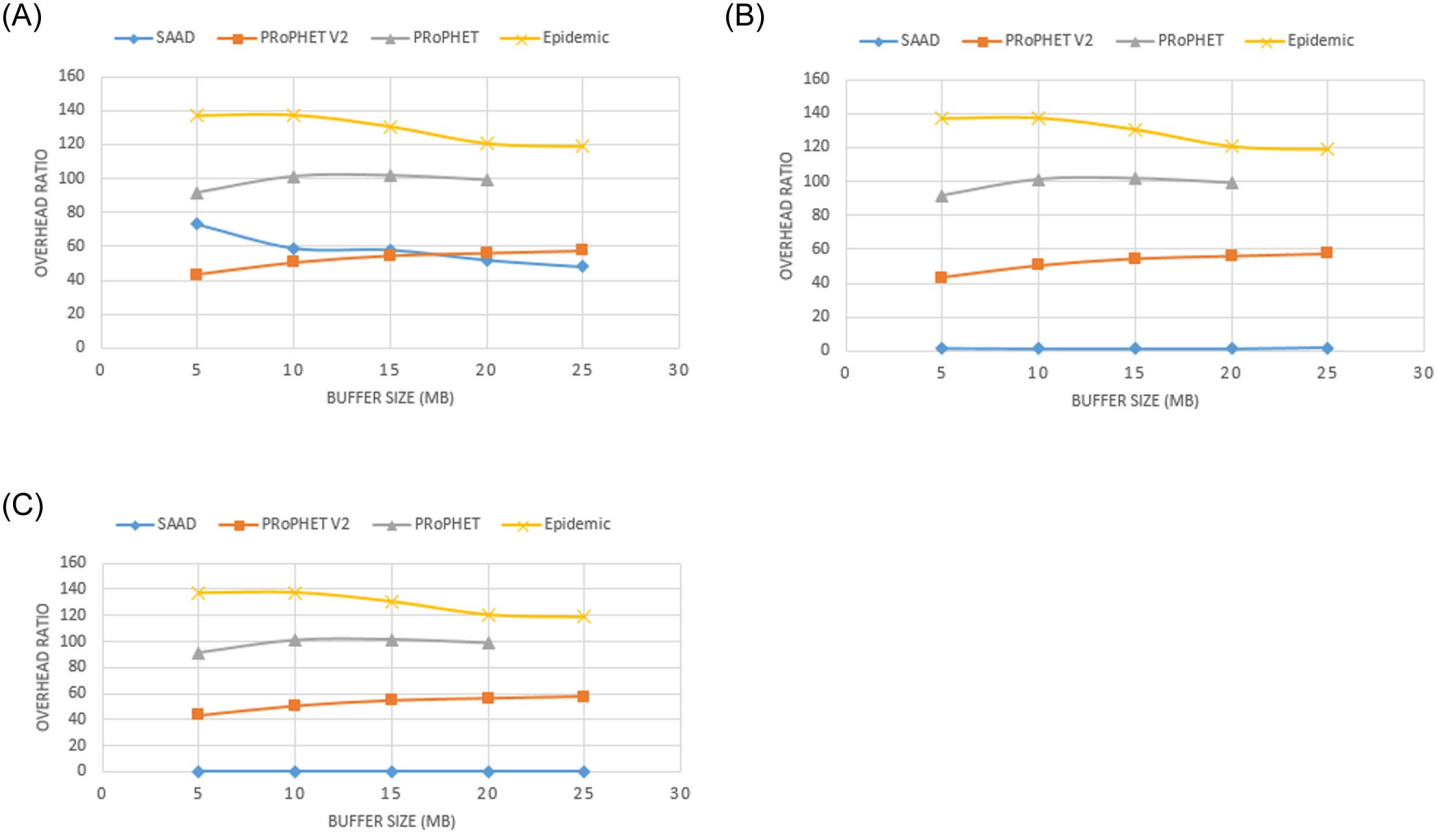
A. Overhead Ratio with different buffer capacity and DC (threshold = 0). B. Overhead Ratio with different buffer capacity and DC (threshold = 2). C. Overhead Ratio with varying buffer size and DC (threshold = 4).

It can be seen in the Fig 6A to 6C that SAAD is delivering more messages than Epidemic, PRoPHET and PRoPHETv2 with lower overhead. When we use threshold value ‘0’, initially SAAD has lower overhead ratio than Epidemic and PRoPHET but higher overhead ratio than PRoPHETv2 as shown in Fig 6A.

However, when we increase the buffer size, the overhead ratio gets lower than PRoPHETv2. When we increase the threshold value to 2, 4 respectively, SAAD uses very less bandwidth and has shown lower overhead as compared to Epidemic, PRoPHET and PRoPHETv2 as shown in Fig 6B and 6C.

Epidemic routing scheme broadcasts the packets to its neighbors. Consequently, a large number of packets are relayed in the network which results in high overhead as shown in Fig 6A to 6C. Unlike Epidemic, PRoPHET forwards packet (s) to those neighboring nodes (may be more than one if they have the same predictability) which meet the given criteria. Therefore, this routing scheme relayed less number of packets as compared to Epidemic. PRoPHETv2 routing scheme refines the predictability formula of PRoPHET to select the popular nodes to forward the packet (s) to the next hop. This routing scheme relayed less number of packets as compared to PRoPHET and Epidemic. However, SAAD forwards packet (s) to only one neighbor node which possesses highest DC. Therefore, our proposed routing scheme relayed very less number of packets as shown in Fig 6A to 6C.

**Fig 7:**
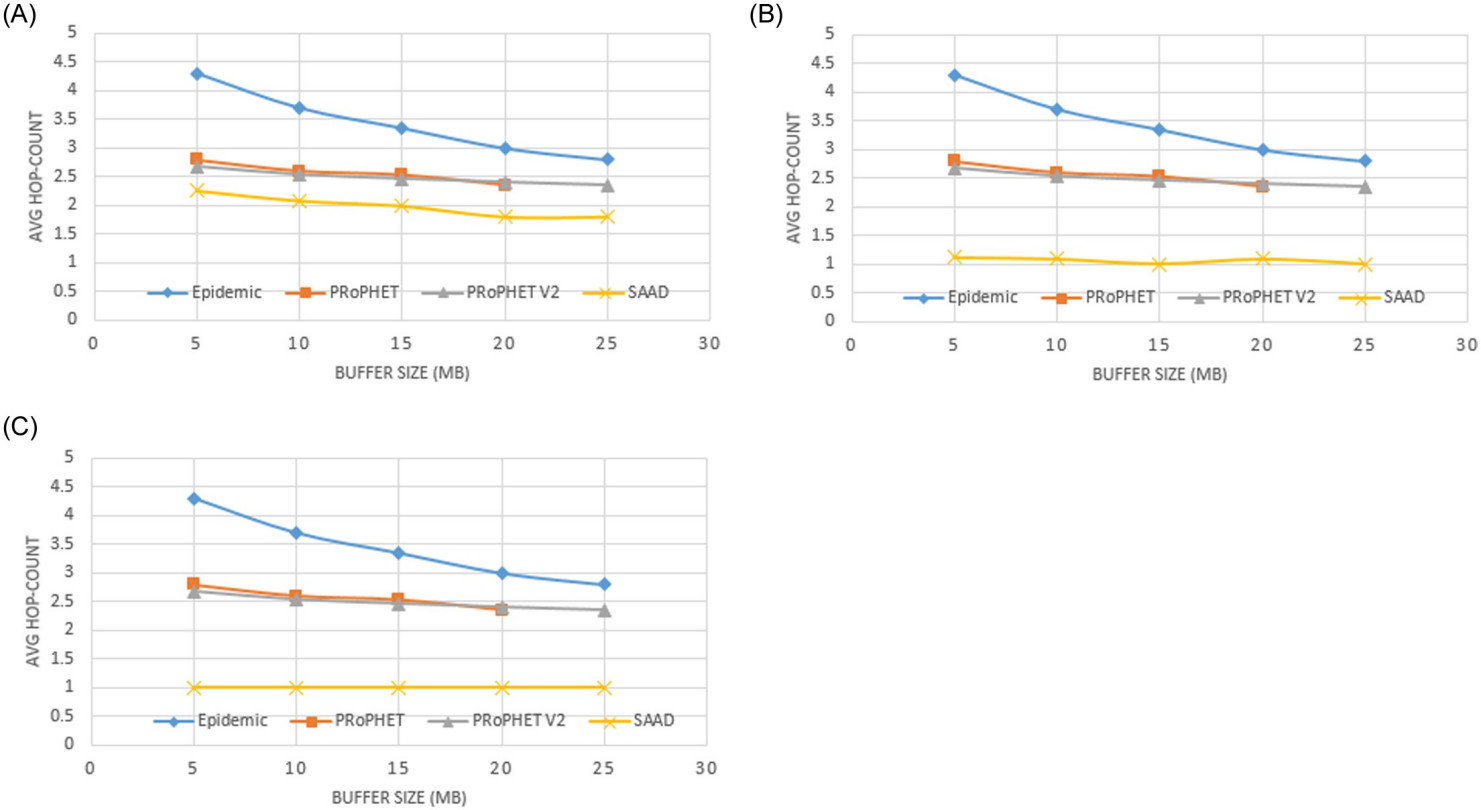
A. Average Hop-count with different buffer capacity and DC (threshold = 0). B. Average Hop-count with different buffer capacity and DC (threshold = 2). C. Average Hop-count with different buffer capacity and DC (threshold = 4).

It can be seen in the Fig 7A to 7C that SAAD is using less number of hops to deliver the packets from the source node to the destination node as compared to Epidemic, PRoPHET and PRoPHETv2. When we use threshold value = ‘0’, Epidemic routing scheme uses highest number of average hop-count, PRoPHET uses slightly more number of average hop-count than PRoPHETv2. However, SAAD uses less average hop-count from 1.5 to 2.3 as shown in Fig 7A. When we use buffer size 5MB, SAAD uses almost 2.3 average hop-count but as we increase the buffer size from 10 MB to 25 MB, the average hop-count gradually decrease from 2.3 to almost 1.5. While when we use threshold value = ‘2’, SAAD uses less number of hops to deliver packets from a source node to the destination node. SAAD uses almost average hop-count 1.3 to 1.6 as shown in Fig 7B. When we use buffer size 5MB 10MB, 15MB, 20MB and 25MB, average hop-count gradually decrease from 1.6 to 1.3. When we use threshold value = ‘4’, SAAD uses only one hop to deliver packets from a source node to the destination node with all buffer sizes (i.e.; 5MB, 10MB, 15MB, 20MB and 25MB) as shown in Fig 7B and 7C.

**Fig 8:**
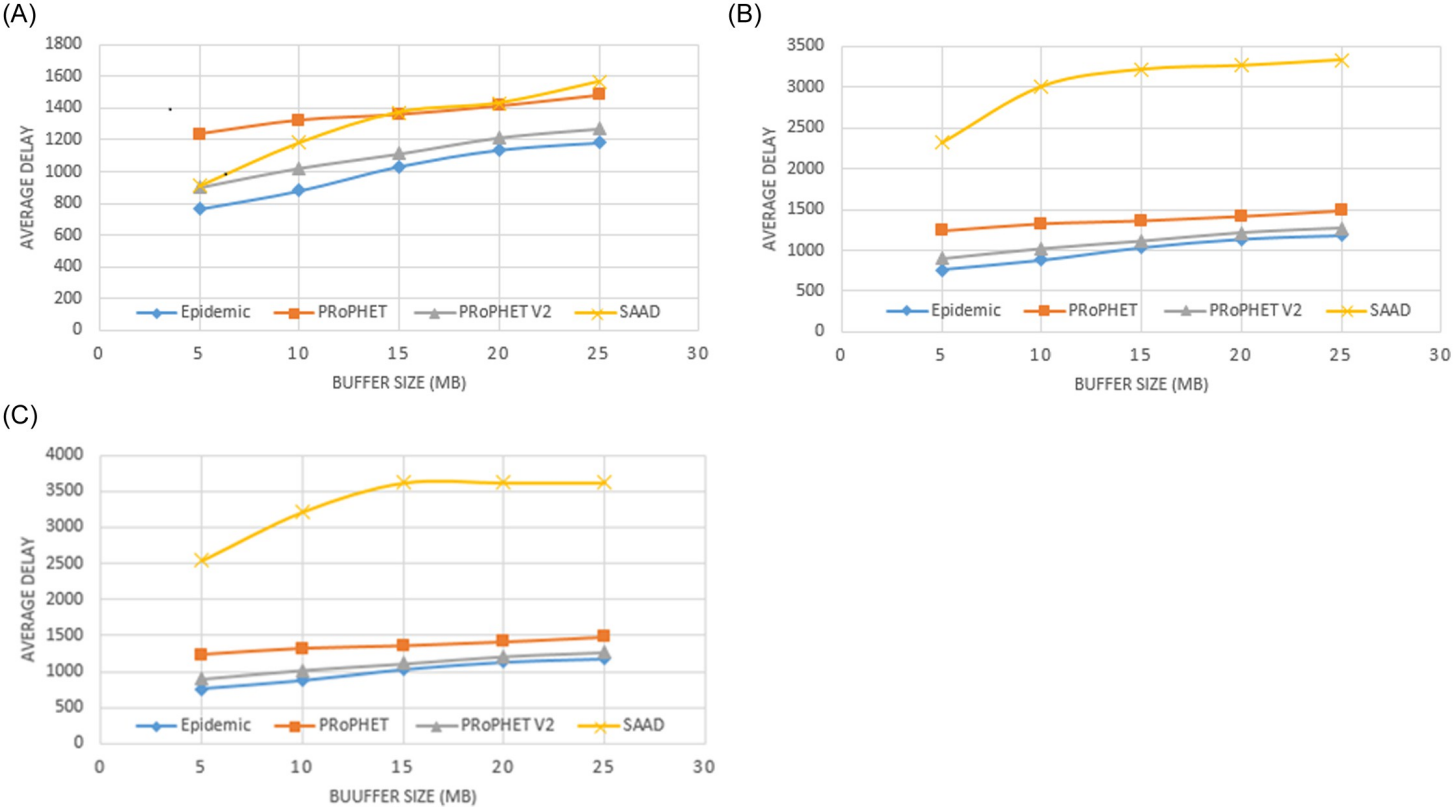
A. Average end-to-end Delay with different buffer capacity and DC (threshold = 0). B. Average end-to-end Delay with different buffer capacity and DC (threshold = 2). C. Average end-to-end Delay with different buffer capacity and DC (threshold = 4).

It can also be seen in the Fig 8A to 8C that SAAD has shown a better improvement as compared to Epidemic, PRoPHET and PRoPHETv2 in terms of delivery ratio, overhead and hop-count but at the cost of higher average end-to-end delay. When we use threshold = ‘0’ (which means even if a node has a single connected node, can participate in forwarding process). Epidemic routing scheme produces the lowest average end-to-end delay. While PRoPHETv2 produces bit higher average end-to-end delay than Epidemic but lower than PRoPHET and SAAD. PRoPHET produces higher average end-to-end delay than Epidemic and PRoPHETv2. However, SAAD initially produces low average end-to-end delay than PRoPHET with buffer size 5 MB and 10 MB, almost the same average end-to-end delay with buffer size 15 MB and 20 MB but, produces higher average end-to-end delay than even PRoPHET when we use buffer size 25 MB as shown in Fig 8A. When we use threshold = ‘2’ and ‘4’, SAAD produces higher average end-to-end delay than Epidemic, PRoPHET and PRoPHETv2 with all buffer sizes (i.e.; 5MB, 10MB, 15MB, 20MB and 25MB) as shown in Fig 8B and 8C.

However, as we increase buffer size, end-to-end average delay of SAAD gradually increases. When we use buffer size 25MB, the end-to-end average delay increases than PRoPHET as shown in Fig 8B. Moreover, when we use threshold 2 or 4, end-to-end average delay increases than Epidemic, PRoPHET and PRoPHETv2 as shown in Fig 8C. Unlike other DTN routing schemes mentioned in section II, SAAD does not make a community group based on same attributes which saves the community cost.

## VII. Conclusion

In this work, we have proposed SAAD routing scheme which uses social attribute (i.e. DC) of a node to calculate the DC of each node. A source node selects forwarder node(s) having DC value greater than a predefined threshold value and itself. The simulation results indicates that SAAD has improved the selection of best forwarder node, delivery ratio, has reduced the overhead and hop-count on the expense of delay as compared to Epidemic, PRoPHET and PRoPHETv2. This work will be extended by exploiting more social metrics to find the more popular forwarder node to transmit packets to the destination node.
